# A systematic seminar-based model improves learning and research competence in an immunology graduate course: a quasi-experimental mixed-methods study

**DOI:** 10.3389/fmed.2026.1787256

**Published:** 2026-04-29

**Authors:** Jing Qin, Wengang Song, Xiujing Feng

**Affiliations:** 1 Shandong Provincial Key Laboratory for Rheumatic Disease and Translational Medicine, The First Affiliated Hospital of Shandong First Medical University and Shandong Provincial Qianfoshan Hospital, Jinan, Shandong, China; 2 School of Clinical and Basic Medical Sciences, Shandong First Medical University & Shandong Academy of Medical Sciences, Jinan, Shandong, China

**Keywords:** Advances in Immunology, higher-order thinking, innovative capacity, seminar-based teaching, teaching methods

## Abstract

**Background:**

Medical immunology, a bridging discipline between basic and clinical medicine, is characterized by rapid knowledge updates, multidisciplinary integration, and fast clinical translation. The course Advances in Immunology aims to bridge the gap between classical theory and cutting-edge exploration, requiring innovative pedagogy to foster critical thinking and research readiness among graduate students. However, traditional lecture-based teaching often fail to develop higher-order competencies. This study designed and applied a systematic seminar-based teaching model for the course Advances in Immunology and evaluated its outcomes.

**Methods:**

A quasi-experimental design with a non-equivalent control group was adopted. Participants were immunology master’s students at Shandong First Medical University (2022 and 2023 cohort: *n* = 38, control; 2024 and 2025 cohort: *n* = 30, intervention). The control group received traditional lecture-based teaching, while the experimental group received the seminar teaching model. Outcomes included academic performance, self-reported competency gains, and qualitative feedback.

**Results:**

Quantitative results showed that the mean final score of the experimental group was significantly higher than that of the control group (93.83 ± 1.97 vs. 90.84 ± 4.35, *p* < 0.001). Questionnaire results indicated significant improvements in literature reading, academic expression, and teamwork skills in the experimental group. Qualitative findings suggested enhanced engagement, higher-order thinking and innovative capacity.

**Conclusion:**

The seminar-based teaching model successfully enhanced academic performance and core research competencies in immunology graduate education, offering a replicable framework for advanced medical courses.

## 1 Introduction

Medical immunology, as an interdisciplinary bridge linking basic and clinical medicine, is characterized by the rapid knowledge updates, interdisciplinary integration, and swift clinical translation (1, 2). In recent years, the advent of emerging technologies, including immune checkpoint inhibitors, CAR-T cell therapy, and innovations like single-cell sequencing, has gradually reshaped the model for diagnosing and treating diseases (3–5).

The course Advances in Immunology aims to bridge the gap between foundational knowledge and frontier exploration. As an advanced course, its core objective is to cultivate innovative talents—in this study defined as graduates who possess the ability to critically evaluate scientific literature, formulate research questions, and design creative solutions to immunological problems, skills essential for independent research and clinical translation. Accordingly, the course also aims to enhance students’ critical literature reading, and develop their innovative thinking and problem-solving abilities (6).

However, traditional lecture-based teaching methods demonstrate limitations when applied to advanced courses (7, 8). Traditional teacher-centered, one-way teaching severely hinders the development of students’ innovative abilities (9, 10). The traditional teaching model mainly has the following limitations: First, student engagement is low, as the classroom is primarily teacher-centered with one-way lectures, leaving students mostly in a passive receiving state, resulting in low participation and a lack of deep interaction (11). For example, research in nursing education shows that traditional lecture-based teaching is difficult to promote the development of students’ critical thinking, with students in traditional classrooms relying more on memorization than on deep understanding (12). Second, the teaching methods are monotonous, making it difficult to accommodate students with different learning styles (13). Finally, classroom time is mainly used for knowledge transmission, with a lack of discussion, practice, and feedback environments, which is unfavorable for the development of higher-order cognitive abilities in students. When it comes to problem-solving and knowledge transfer, the effectiveness of traditional lectures is markedly inferior to that of interactive, student-centered teaching (11, 13). As a result, students may graduate with a superficial grasp of complex immunological concepts and insufficient preparedness for independent research or clinical problem-solving (14).

Modern higher education is gradually transitioning from teacher-centered teaching to student-centered, competency-oriented teaching (15–17). The seminar-based teaching model, originating from the University of Berlin in the 19th century, adheres to the principle of “integration of teaching and research” and has gained wide attention (18). The seminar-based teaching model is conducted in small group discussions, emphasizing teachers and students as “co-researchers,” aligning with constructivist and collaborative learning theories (19, 20). Although some studies report positive outcomes of seminar-based teaching models in medical education (21), existing research still has two limitations: most applications focus on isolated teaching sessions and lack systematic, process-oriented instructional design, and there is a lack of mixed methods combining quantitative and qualitative verification (22, 23).

To address these gaps, we developed a systematic seminar-based teaching model for Advances in Immunology and evaluated its effects using a non-randomized controlled quasi-experimental design with mixed methods. By integrating quantitative measures of academic performance with qualitative insights from surveys, interviews, and classroom observations, this study provides both empirical evidence of the model’s effectiveness and a detailed framework for implementation. Our findings offer theoretical support and practical guidance for cultivating high-level, innovative talents in immunology and other advanced biomedical disciplines.

## 2 Methods

### 2.1 Study design and participants

This study adopted a non-equivalent control group quasi-experimental design. This study did not use individual randomization because “Advances in Immunology” is a core required course for master’s students in immunology and must be scheduled according to consecutive academic years. Splitting the class is impractical and would violate teaching ethics. Therefore, a natural class cluster design was adopted, with the 2022/2023 academic year class serving as the control group and the 2024/2025 academic year class as the experimental group. The baseline characteristics of the two groups of students are comparable, and the same teacher teaches both groups according to a unified syllabus, which ensures the teaching order and maintains a real classroom environment while minimizing the bias caused by non-randomized grouping.

The study participants were master’s students in immunology in the 2022, 2023, 2024 and 2025 grades of Shandong First Medical University. Students in the class of 2024 and 2025 (*n* = 30) will receive a systematic seminar teaching model as the experimental group. Students in the class of 2022 and 2023 (*n* = 38) were used as a control group to receive traditional lecture-style teaching. The master’s program is a three-year program. Advances in Immunology is a first-year, first-semester core course (1 credit, 16 contact hours). All students were enrolled in the same set of concurrent core courses, including molecular biology techniques and medical statistics, during the same semester to ensure comparable academic workload across cohorts. All participants had completed foundational coursework in immunology and related disciplines during their undergraduate studies, with no significant differences observed between the control and experimental groups. This ensured baseline comparability in prior immunology-related training. The two groups of students are comparable in terms of basic characteristics such as age and core professional courses taken.

This research plan was approved by the Ethical Review Committee of Shandong First Medical University [Approval Number: (R202601120005)]. All teaching and assessment activities are regular course teaching sessions, and the anonymized grades and questionnaire data involved are used for educational research, which has been explained to all participants, and informed consent has been obtained.

### 2.2 Interventions

Both the experimental and control groups were enrolled in the same course, *Advances in Immunology* (a 1 credit, 16-h course), and were taught by the same main lecturer. The use of textbooks and core syllabus was consistent to ensure the comparability of other major teaching variables except the teaching mode.

#### 2.2.1 Control group (class of 2022 and 2023)

The course consisted of eight weekly 90-min lectures covering the same six core topics as the experimental group (Table 1). Each lecture was delivered by the instructor using PowerPoint slides and clinical case examples, with limited student interaction. Students were assigned the same core literature package but did not participate in presentations or peer-led discussions. A detailed weekly schedule of lecture content and learning objectives is provided in Supplementary Table S1.

**Table 1:** Seminar topic design for the course Advances in Immunology.

No.	Seminar theme	Related disciplines	Expected seminar objectives
1	Immune-related adverse events and coping strategies of immune checkpoint inhibitors in cancer (32–34)	Oncology, pharmacology	Understand resistance mechanisms and propose combination strategies
2	Challenges and optimization of CAR-T cell therapy in solid tumors (4, 35, 36)	Cell biology, clinical medicine	Analyze therapeutic bottlenecks and design optimization approaches
3	Role of metabolic reprogramming in infection and autoimmune diseases (37–39)	Metabolic biology, infectious diseases	Grasp metabolic regulatory mechanisms and explore novel therapeutic targets
4	Advances of single-cell technologies in immunological research (5, 40, 41)	Bioinformatics, pathology	Understand technical principles and evaluate application value
5	Mechanisms of gut microbiota-immune system interactions and their clinical implications (42–44)	Microbiology, gastroenterology	Analyze regulatory pathways and discuss potential intervention strategies
6	Applications of artificial intelligence (AI) in immunology research and clinical translation (45–47)	Computer science, clinical medicine	Understand AI application scenarios and reflect on ethical considerations

#### 2.2.2 Experimental group (class of 2024 and 2025)

Implemented the systematic seminar-based teaching model constructed in this study, which included four modules (see below for specific construction).

##### 2.2.2.1 Teaching objective reconstruction

To align with the seminar-based teaching model, the course objectives were restructured according to a three-dimensional framework of “knowledge–ability–competence”:

Knowledge: Mastering cutting-edge topics and core technologies.
Ability: Literature retrieval, critical reading, scientific questioning, and teamwork.
Competence: Lifelong learning, independent problem-solving, and research innovation.

This framework guided the design of the seminar activities and the development of the evaluation instruments.

##### 2.2.2.2 Collaborative preparation between teachers and students

###### 2.2.2.2.1 Teacher role

The instructor systematically and comprehensively introduced the development and frontiers of the discipline, selected six topics covering classics, cutting-edge, and clinical (Table 1); and provided a “core literature package” for each topic. The instructor explained the rules of the seminar through the introductory class and provided methodological guidance during the seminar.

###### 2.2.2.2.2 Student preparation

Adopting the “group cooperation and individual division of labor” mode, take the group as a unit, under the guidance of the teacher, conduct an extended literature search, carry out in-depth reading and analysis, and jointly prepare a structured presentation. The presentation materials are submitted 48 h prior to the seminar and shared with the entire class.

#### 2.2.3 Course structure and organization

The course, “Advances in Immunology,” was a first-year, first-semester core course worth 1 credit, with a total of 16 contact hours. The course met once a week for 4 h per session over 4 weeks. The experimental group consisted of 30 students, while the control group comprised 38 students.

Students in the experimental group were divided into six groups, with 4–6 members per group, each assigned to one of the six seminar themes (Table 1). Each group presented once during the semester, and group composition remained consistent throughout the course. This structure ensured that all students had the opportunity to participate in in-depth preparation and presentation while maintaining stable collaborative relationships.

#### 2.2.4 Weekly seminar workflow

Each seminar topic (Table 1) is scheduled over one week. The workflow is structured as follows:

Instructor preparation: One week before the seminar, the instructor provides a “core literature package” (3–5 key papers) to all students via the course platform.
All-student preparation: Every student is required to read the core literature and identify key concepts, controversies, and potential discussion points.
Presenting group: A student group (4–5 members) is assigned to the topic. This group conducts an extended literature search, critically synthesizes the findings, and prepares a structured presentation. The presentation slides are submitted 48 h prior to the seminar and made available to the entire class.
Non-presenting students: They read both the core literature and the presentation materials prepared by their peers, and prepare at least one analytical question or comment to contribute to the interactive discussion.
In-class session: The session follows the “three-step progressive” structure (theme report → interactive discussion → summary and sublimation), with active participation from all students.
Theme report (30 min): After the teacher introduces the topic, the group members will report and accept the evaluation of other group members and teachers.
Interactive discussion (45 min): Supplementary elaboration, debate, and in-depth discussion between groups around the topic of the report.
Summary and sublimation (15 min): The instructor comprehensively summarized core ideas, supplement frontiers, and offered feedback.
End-of-term review paper: At the conclusion of the seminar series, each student independently selects a topic of personal interest and writes a 3,000–5,000 word review paper, drawing upon the literature and insights acquired throughout the course.

This workflow ensures that all students engage deeply with each topic, while the presenting group takes responsibility for in-depth synthesis and peer learning. The end-of-term review paper allows students to explore a topic of their choice in greater depth.

#### 2.2.5 Diversified evaluation system

Combination of procedural evaluation and final evaluation: 60% of the procedural evaluation (30% for group reports, 20% for classroom participation, and 10% for literature review); the final evaluation accounts for 40% (30% for the final thesis and 10% for the oral defense). Establish a three-level feedback mechanism for immediate feedback (classroom review), stage feedback (summary every 4 weeks), and final feedback (total grade analysis and personalized suggestions) to help students continue to improve.

#### 2.2.6 Academic performance assessment

For the control group, assessment was based on a final written exam (100%). For the experimental group, the diversified evaluation system described above was implemented. To enable a valid comparison of academic performance between the two groups, the final exam component was extracted from both groups’ overall grades. The final exam was identical for both groups, consisting of the same written questions and scoring rubrics. The statistical comparisons reported in Tables 2, 3 are therefore based solely on this common assessment instrument.

**Table 2:** Descriptive statistics of course grades between the two groups.

Statistical indicator	Control group (*n* = 38)	Experimental group (*n* = 30)	Difference (experimental − control)
Mean ± SD	90.84 ± 4.35	93.83 ± 1.97	+2.99 ± 2.66
Median	91.0	93.0	+2.0
Interquartile range (IQR)	4.75	3.0	−1.75
Minimum (Min.)	80.0	91.0	+11.0
Maximum (Max.)	98.0	98.0	0.0

Data are presented as mean (standard deviation), median (interquartile range), minimum and maximum. The experimental group exhibited higher central tendency and less score dispersion than the control group.

**Table 3:** Results of inferential statistical tests comparing course outcomes between groups.

Outcome/comparison	statistical test applied	Test statistic	*p*-value	Effect size (95% CI)
Difference in mean scores	Independent samples *t*-test (Welch’s)	*t* = 3.78	<0.001	Cohen’s *d* = 0.92, 95% CI: 0.45 to 1.38
Difference in score distributions	Mann–Whitney *U* test	*U* = 318.0	<0.01	*r* = 0.38
Difference in excellence rate	Chi-square test	*χ*^2^ = 8.3353	<0.01	Cramér’s *V* = 0.35

Welch’s *t*-test was applied due to unequal variances (Levene’s test, *p* = 0.005). The excellence rate was based on the percentage of students scoring ≥90. Effect sizes are reported with 95% confidence intervals (CI) where applicable.

### 2.3 Survey instrument: design and validation

To evaluate the practical effectiveness of the seminar teaching model, this study developed a self-designed “Seminar Teaching Model Effectiveness Questionnaire” and administered it to the experimental group. The questionnaire was designed specifically for the seminar-based model (e.g., group collaboration, literature review writing, interactive discussion) and was therefore administered only to the experimental group.

The questionnaire consisted of 17 items, including 14 Likert-scale items (5-point scale, from 1 = “strongly disagree” to 5 = “strongly agree”) and 3 multiple-choice/open-ended questions for qualitative feedback. The Likert-scale items were grouped into four dimensions: (1) learning initiative (3 items, Q6–Q8); (2) skill improvement (4 items, Q9–Q12); (3) course design and implementation (5 items, Q13–Q17); and (4) overall satisfaction (2 items, Q18 and Q22). The questionnaire aimed to systematically assess teaching effectiveness from cognitive, behavioral, and emotional perspectives. The full questionnaire is provided as Supplementary Table S2.

Reliability was assessed using Cronbach’s *α* coefficient, and construct validity was examined through exploratory factor analysis (EFA) with principal component extraction and varimax rotation. The criteria for validity included a Kaiser–Meyer–Olkin (KMO) measure greater than 0.70, a significant Bartlett’s test of sphericity (*p* < 0.05), and factor loadings above 0.40.

After the course, the questionnaire was distributed to the experimental group (*n* = 30). Quantitative data were analyzed using descriptive statistics (mean and standard deviation), reporting the mean and standard deviation for each item and dimension; open-ended responses were subjected to thematic analysis to extract core perspectives and feedback trends from the student experience.

### 2.4 Data collection and analysis

A mixed-methods approach was employed.

#### 2.4.1 Classroom observations

Classroom observations were conducted using the AI Classroom Quality Analysis System of the Leke Teaching Cloud Platform during three randomly selected sessions per group. The system automatically tracked the frequency of active contributions (via speech detection and speaker identification), classified question types into analytical/innovative versus factual using natural language processing, and measured collaborative behaviors such as turn-taking frequency, discussion duration, and discourse patterns (e.g., building on others’ ideas, clarifying, providing feedback). Data from the AI system were analyzed using frequency counts (for active contributions and question types) and thematic categorization (for discourse patterns and collaborative behaviors).

#### 2.4.2 Quantitative data

Quantitative data were analyzed using SPSS (Version 27.0). Descriptive statistics are presented as means with standard deviations (SD) and medians with interquartile ranges (IQR). The normality of data distribution for each group was assessed using the Shapiro–Wilk test. Due to significant heteroscedasticity between groups (Levene’s test, *p* = 0.005), a Welch’s independent samples *t*-test was used to compare mean scores, with Cohen’s *d* as the effect size measure. Given the non-normal distribution in one group, the nonparametric Mann–Whitney *U* test was also conducted to compare score distributions, with effect size reported as *r* = |*Z*|/√*N*. The proportion of students achieving excellence (scores ≥90) was compared using a chi-square test with Yates’ continuity correction, and Cramér’s *V* was reported as the effect size. Effect sizes are reported with 95% confidence intervals where applicable. All statistical tests were two-tailed, with a significance level set at *p* < 0.05.

#### 2.4.3 Qualitative responses

Qualitative responses from open-ended questions were categorized and analyzed thematically.

## 3 Results

Quantitative results showed that the proportion of excellent grades in the experimental group was significantly higher than that in the control group. Questionnaire results indicated significant improvements in literature reading, academic expression, and teamwork skills in the experimental group. Qualitative results demonstrated that the seminar enhanced students’ higher-order thinking and innovation capabilities.

### 3.1 Academic performance

To evaluate the effectiveness of the seminar-based teaching reform, final course scores of the two groups were collected from the experimental group (2024 and 2025 Immunology Master’s Class, *n* = 30) and the control group (2022 and 2023 Class, *n* = 38) who received traditional instruction. A mixed-methods analytical approach was employed, encompassing both descriptive and inferential statistics. The descriptive characteristics of the final course grades for both groups are summarized in Table 2. The data showed that the experimental group attained a markedly higher mean score (93.83 ± 1.97) compared to the control group (90.84 ± 4.35). Notably, the standard deviation in the experimental group was less than half of that in the control group (1.97 vs. 4.35), indicating substantially more consistent performance among students exposed to the new teaching model. This pattern of reduced score dispersion is further reflected in the interquartile ranges (IQR): the experimental group’s scores were concentrated within a narrow band of 92.00–95.00 (IQR = 3.00), whereas the control group’s scores were spread across a wider range of 89.00–93.75 (IQR = 4.75). Furthermore, when defining excellence as a score of 90 or above, all students in the experimental group met this criterion (100%, 30/30), compared to 86.84% (33/38) in the control group.

**Figure 1:**
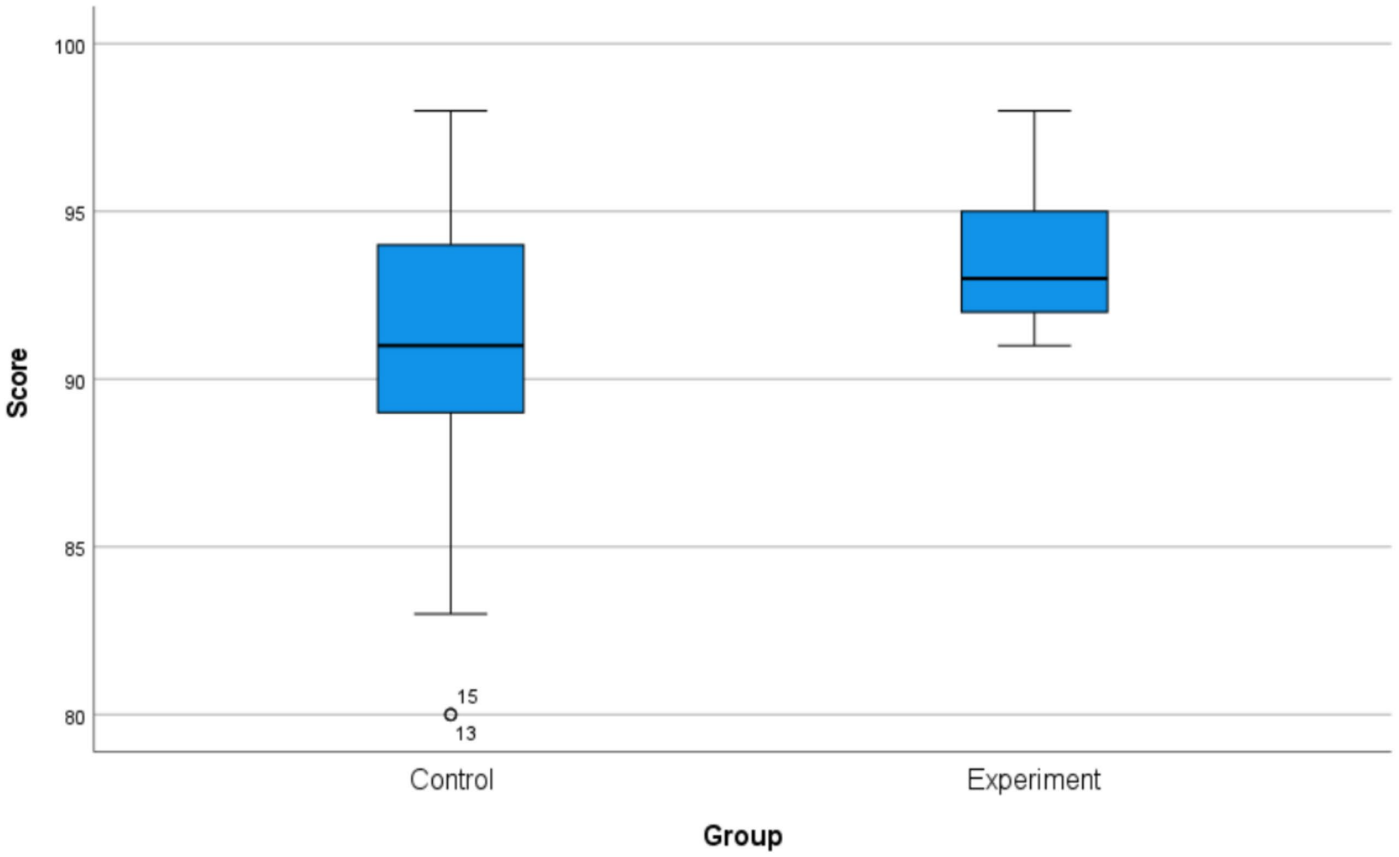
Comparison of final exam scores between the control and experimental groups.

The results of the statistical comparisons, presented in Table 3, confirm that the observed differences are statistically significant and educationally meaningful. Due to significant heteroscedasticity between the groups [Levene’s test, *F*(1, 66) = 8.53, *p* = 0.005], a Welch’s independent samples *t*-test was conducted. This test revealed a highly significant difference in mean scores [*t*(54) = 3.78, *p* < 0.001], with a large effect size (Cohen’s *d* = 0.92, 95% CI: 0.45 to 1.38). This finding was corroborated by a non-parametric Mann–Whitney *U* test, which also indicated a significant difference in the overall score distributions (*U* = 318.0, *r* = 0.38, *p* = 0.0014). Furthermore, a chi-square test confirmed that the excellence rate (≥ 90 points) was significantly higher in the experimental group (*χ*^2^ = 8.335, *p* = 0.004), with a moderate-to-large effect size (Cramér’s *V* = 0.35). Collectively, these results demonstrate that the experimental group achieved superior academic outcomes across all measured dimensions (see Figure 1).

The box plot depicts the distribution of final scores for the control group (traditional lecture-based teaching, *n* = 38) and the experimental group (seminar-based teaching model, *n* = 30). The central line within each box represents the median, the box boundaries indicate the interquartile range (IQR; 25th to 75th percentiles), and the whiskers extend to the minimum and maximum values within 1.5 × IQR. Individual data points are shown. The Mann–Whitney *U* test indicated that the score distribution of the experimental group was significantly higher than that of the control group (*p* = 0.0014).

### 3.2 Seminar-based teaching effect Questionnaire

#### 3.2.1 Reliability and validity analysis

##### 3.2.1.1 Reliability

Cronbach’s *α* coefficients for the four dimensions were 0.929, 0.927, 0.802, and 0.911, respectively, all exceeding the acceptable threshold of 0.70. The overall *α* coefficient for the questionnaire was 0.867. These results indicate good internal consistency reliability.

##### 3.2.1.2 Construct validity

Exploratory factor analysis extracted four factors (KMO = 0.713, Bartlett’s test *p* < 0.001), accounting for 85.26% of the total variance, with factor loadings ranging from 0.659 to 0.922. The factor structure aligned well with the theoretical framework.

#### 3.2.2 Descriptive statistics of the seminar-based teaching effectiveness questionnaire

The Likert-scale items (14 items) were scored on a 5-point scale. As shown in Table 4, the average scores of the experimental group across all three questionnaire dimensions exceeded 4.0, with the highest score observed in overall satisfaction (4.37 ± 0.89). Pearson correlation analysis indicated that the total questionnaire score and the ability enhancement dimension score were significantly positively correlated with the final course grade (*r* = 0.326, *p* < 0.05; *r* = 0.351, *p* < 0.05).

**Table 4:** Scores of the seminar-based teaching effectiveness questionnaire in the experimental group.

Dimension	Item number	Average score
Learning initiative and interest	3 (Q6–Q8)	4.23 ± 0.82
Ability enhancement	4 (Q9–Q12)	4.18 ± 0.73
Course design and implementation	5 (Q13–Q17)	4.29 ± 1.03
Overall satisfaction	2 (Q18, Q23)	4.37 ± 0.89
Total score	20	4.25 ± 0.89

Data are presented as mean ± standard deviation (SD). Responses were measured on a 5-point Likert scale (1 = strongly disagree, 5 = strongly agree).

### 3.3 Classroom observation

The experimental group demonstrated greater levels of classroom engagement than the control cohort. The average number of active contributions per class was 18.6, while the control group made only 5.2 contributions per class. The experimental group asked analytical and innovative questions, which made up 65% of their total queries, while the control group asked these types of questions at only 22%. Actively collaborative behaviors in the experimental group (such as sharing materials and coordinating work) accounted for 82% of total collaborative behaviors, significantly higher than 35% in the control group.

### 3.4 Qualitative results

#### 3.4.1 Interviews

##### 3.4.1.1 Student interviews

Based on interviews, students generally reported that the seminar-based teaching transformed them from passive recipients of knowledge into active explorers, enabling them to gain a deep understanding of cutting-edge directions in immunology. Master’s student A stated, “After reading literature, reporting, and discussing throughout the whole process, I am no longer just memorizing knowledge. I have learned to think like a researcher, to identify research highlights, and to focus on scientific questions. My research abilities have greatly improved, and this training is very helpful for both posing scientific questions and writing academic papers.” Master’s student B noted, “The open classroom atmosphere encouraged me to express my ideas more confidently. When I felt very nervous during my first presentation, the encouragement of teachers and classmates gradually made me more willing to share my opinions.” Master’s student C expressed, “Group collaboration and personalized guidance from the instructor helped me quickly understand cutting-edge knowledge, and my learning efficiency has significantly improved compared to before. Group collaboration not only reduces individual pressure but also enhances the academic atmosphere.” However, some students also presented several challenges: first, significant time pressure because cutting-edge literature is difficult and time-consuming, and with regular lab duties, sometimes it is hard to fully prepare for seminars; second, a lack of understanding of some advanced technologies, such as the principles of single-cell sequencing, posed a significant barrier to their in-depth participation in academic discussions.

##### 3.4.1.2 Teacher interviews

Teachers believe that the seminar model aligns well with the teaching needs of the course Advances in Immunology, effectively achieving the three-dimensional goal of “knowledge-ability-competence.” One teacher stated, “The seminar-based teaching model can effectively make up for the shortcomings of the traditional classroom, allowing students to become the main agents of knowledge construction. Under the guidance of teachers, students autonomously search for literature, learn to critically read papers, and raise scientific questions. It teaches students how to obtain academic information and to pose questions critically, which is exactly at the core of cultivating innovative talents.”

From the perspective of the entire process, students’ academic expression and logical thinking abilities have significantly improved. By reading literature and writing reviews, they have laid a solid foundation for writing research papers in the future. At the same time, as teachers, the required skills are substantial, and there are some challenges. Unlike the traditional model, where preparing lessons mainly by using textbooks sufficed, the seminar model requires teachers to structure knowledge and provide guidance in a more dynamic and facilitative way, thus placing higher demands on instructional design and facilitation skills.

### 3.5 Case analysis

At the seminar “Challenges and Optimization of CAR-T Cell Therapy in Solid Tumors” a student research team put forward an integrated solution that combines bispecific CAR engineering with targeted gene editing and methods for tumor microenvironment modulation. This strategy addresses fundamental challenges in treating solid tumors, including the difficulty of CAR-T cell infiltration and significant side effects in other body parts. The team successfully handled various questions during the Q&A session while they presented their proposal through logical reasoning and practical evidence. Ultimately, the group’s seminar results were selected for presentation at the university-level academic forum. This practical case fully demonstrates that the seminar teaching model has significant effectiveness in cultivating students’ translational medicine thinking and research innovation abilities.

## 4 Discussion

This study validates the efficacy of a systematic seminar-based teaching model designed for advanced immunology courses, which has been shown to enhance the academic performance and research competency of master’s students in immunology—findings that align with previous findings (19, 24–27).

Seminar-based teaching has been demonstrated to significantly enhance students’ case analysis skills and learning satisfaction (19), while also improving overall assessment performance, including theoretical knowledge acquisition (26). Additionally, this pedagogical approach has been shown to be instrumental in cultivating higher-order cognitive abilities, such as critical thinking (25), and yields optimal outcomes within inclusive, interactive learning environments (27). Collectively, these findings indicate that the seminar-based teaching model is effective in facilitating the integration of theoretical knowledge with clinical practice and in promoting deep learning.

More importantly, the study introduces an integrative framework incorporating three-dimensional objective reconstruction and a three-stage process design. To validate this framework, the research employed a non-equivalent control group experiment design, paired with a mixed-methods approach that combines quantitative metrics and qualitative analytical techniques, thus contributing new insights to the existing body of literature.

### 4.1 Key innovations

This study introduces a systematic seminar-based model structured around four integrated phases: objective setting, pre-class preparation, classroom implementation, and diversified assessment. Unlike previous interventions that often focus on isolated teaching components, this framework establishes a coherent, comprehensive learning cycle in which each phase deliberately aligns with and informs the subsequent phase.

This design overcomes a key limitation in prior literature, which frequently treated seminar teaching as an independent activity rather than as part of a sequential pedagogical process (28–30). Furthermore, the adoption of a mixed-methods approach—integrating quantitative performance data with qualitative insights from observations, surveys, and interviews—facilitates a more nuanced and rigorous evaluation. This triangulation not only captures changes in academic outcomes but also elucidates the mechanisms underlying these changes, providing a more comprehensive understanding of the educational process than could be achieved through either method alone (29, 31). Ultimately, the model is designed to systematically strengthen core research competencies. By engaging students in critical literature analysis, structured discussion, and collaborative problem-solving, it encourages a shift from passive knowledge acquisition to active researcher development. Consequently, students demonstrate enhanced abilities in synthesizing literature, exercising critical thought, and working in teams—key advancements that improve their preparedness for independent research and future clinical practice.

### 4.2 Key success factors

The success of the seminar-based model depends on three key factors: Transformation of the teacher’s role from “knowledge transmitters” to “designers and facilitators” of the classroom, necessitating a solid disciplinary foundation, adept classroom management skills, and a keen awareness of disciplinary advancements. This aligns with the social constructivist perspective, where the core role of the teacher is to assist students in collectively constructing knowledge. A well-established structured support system, comprising the selection of scientific topics, provision of high-quality literature resources, reasonable group allocation, and a diverse evaluation system, can significantly reduce students’ cognitive load, enabling them to engage in higher-order thinking activities. Creating an open and inclusive classroom atmosphere that encourages students to question boldly, express actively, and respect diverse viewpoints and academic opinions.

### 4.3 Limitations of the study

The research contains some restrictions: First, the sample size was small and drawn from the same program at a single university, which may affect the generalizability of the results. Future studies need to repeatedly validate these findings through more studies and different programs. Second, the evaluation of advanced competencies depends mainly on student personal accounts and teacher-based observations. Future studies should use more objective standardized assessment tools, which provide objective measurements and workplace performance evaluations for evaluation purposes. Finally, the research examined teaching effects during one semester, but the impact of the seminar-based model on students’ academic performance in the future remains unclear. Future research should implement quantitative assessment methods and long-term follow-up for an extended period.

## 5 Conclusion

The systematic seminar-based teaching model developed in this study, designed around the three-dimensional objectives of “knowledge–ability–competence” and the three-stage process of “presentation–discussion–sublimation,” effectively improved the teaching quality of the course Advances in Immunology. The model significantly enhanced students’ academic performance and cultivated core competencies in literature analysis, academic expression, teamwork, and scientific innovation, establishing a teaching ecosystem of “teacher-student collaborative inquiry” and promoting mutual growth in teaching and learning.

The research findings suggest that the systematic seminar teaching model is an effective approach for teaching reform in advanced medical courses and can offer insights for teaching practices in other cutting-edge courses.

## Data Availability

The original contributions presented in the study are included in the article/Supplementary material, further inquiries can be directed to the corresponding author.
