# Supplementary material for: A systematic seminar-based model improves learning and research competence in an immunology graduate course: a quasi-experimental mixed-methods study
Source: Front Med (Lausanne). 2026 Apr 29;13:1787256. doi: 10.3389/fmed.2026.1787256 (PMC13169503; doi:10.3389/fmed.2026.1787256)
Supplement: Supplementary file 1 [file Table_1.docx]

**Table S1. Unified Course Syllabus and Learning Objectives Across Both Teaching Groups**

| No. | Topic | Lecture Content & Key Points | Learning Objectives |
| --- | --- | --- | --- |
| 1 | Immune-related Adverse Events and Coping Strategies of Immune Checkpoint Inhibitors in Cancer | Overview of immune checkpoint pathways; clinical case studies of irAEs; management guidelines | Understand resistance mechanisms and propose combination strategies |
| 2 | Challenges and Optimization of CAR-T Cell Therapy in Solid Tumors | CAR-T design principles; barriers in solid tumors (TME, trafficking); emerging engineering solutions | Analyze therapeutic bottlenecks and design optimization approaches |
| 3 | Role of Metabolic Reprogramming in Infection and Autoimmune Diseases | Metabolic pathways (glycolysis, OXPHOS); immunometabolism in T cells and macrophages | Grasp metabolic regulatory mechanisms and explore novel therapeutic targets |
| 4 | Advances of Single-cell Technologies in Immunological Research | scRNA-seq, scATAC-seq principles; data analysis pipelines; applications in immunology | Understand technical principles and evaluate application value |
| 5 | Mechanisms of Gut Microbiota–Immune System Interactions and Their Clinical Implications | Microbiota composition; immune modulation mechanisms (SCFAs, bile acids); therapeutic interventions | Analyze regulatory pathways and discuss potential intervention strategies |
| 6 | Applications of Artificial Intelligence (AI) in Immunology Research and Clinical Translation | AI/ML basics; predictive modeling in immuno-oncology; ethical considerations | Understand AI application scenarios and reflect on ethical considerations |
| 7 | **Review and Integration** | Summary of key concepts across all topics; discussion of cross-cutting themes; case-based review | Synthesize knowledge and identify connections between topics |
| 8 | **Final Assessment and Course Wrap-up** | Final exam review; feedback session; course evaluation | Consolidate learning and reflect on course outcomes |

Note: The same weekly topics, core content, and learning objectives were delivered to both the control group (via traditional lectures with identical reading assignments) and the experimental group (via seminar-based discussions). Both groups completed the same final assessment.
