# Supplementary material for: A systematic seminar-based model improves learning and research competence in an immunology graduate course: a quasi-experimental mixed-methods study
Source: Front Med (Lausanne). 2026 Apr 29;13:1787256. doi: 10.3389/fmed.2026.1787256 (PMC13169503; doi:10.3389/fmed.2026.1787256)
Supplement: Supplementary file 2 [file Table_2.docx]

Survey Questionnaire on Seminar Teaching Effectiveness in Immunology.docx

Basic Information

1. Your role:

□ Undergraduate student (Clinical Medicine/Basic Medicine)

□ Master's student

□ Doctoral student

□ Faculty

□ Other (Please specify) ______

1. Your field(s) of study (multiple selections allowed):

□ Clinical Medicine (Senior undergraduate/Graduate level)

□ Basic Medicine (Senior undergraduate/Graduate level)

□ Immunology

□ Other ______

1. Number of Seminars you have participated in:

□ 1-2 times

□ 3-5 times

□ More than 5 times

1. Your role(s) within the Seminar group (multiple selections allowed):

□ Literature search and organization

□ Core idea extraction

□ PowerPoint presentation creation

□ Main presenter

□ Q&A responder

□ Discussion recorder

1. Do you have a background in immunology-related research?

□ Yes (Please specify field: ______)

□ No

II. Learning Initiative and Interest

1. Compared to traditional lectures, does the Seminar model make you more proactive in consulting cutting-edge immunology literature?

□ Strongly disagree

□ Disagree

□ Neutral

□ Agree

□ Strongly agree

1. Do you proactively follow the latest research developments (e.g., top-tier journal papers, academic conferences) related to the Seminar topics?

□ Strongly disagree

□ Disagree

□ Neutral

□ Agree

□ Strongly agree

1. Does the Seminar model enhance your interest in learning about frontier areas of immunology?

□ Strongly disagree

□ Disagree

□ Neutral

□ Agree

□ Strongly agree

III. Effectiveness in Skill Enhancement

Does the Seminar help you improve the following skills? (Single choice, rate 1-5)

1. Literature search and screening skills (e.g., accurately finding papers from top-tier journals)

□ No improvement

□ Slight improvement

□ Moderate improvement

□ Significant improvement

□ Very significant improvement

1. Critical reading skills (e.g., analyzing flaws in literature)

□ No improvement

□ Slight improvement

□ Moderate improvement

□ Significant improvement

□ Very significant improvement

1. Academic presentation skills (e.g., clearly reporting findings)

□ No improvement

□ Slight improvement

□ Moderate improvement

□ Significant improvement

□ Very significant improvement

1. Team collaboration skills (e.g., dividing tasks and cooperating with group members)

□ No improvement

□ Slight improvement

□ Moderate improvement

□ Significant improvement

□ Very significant improvement

IV. Evaluation of Course Design and Implementation

1. Are the Seminar topics closely aligned with cutting-edge advances in immunology?

□ Very closely aligned

□ Closely aligned

□ Moderately aligned

□ Slightly aligned

□ Not aligned at all

1. Does the depth and breadth of the presentation content meet your learning needs?

□ Both depth and breadth are sufficient

□ Depth and breadth are mostly sufficient

□ Sufficient depth but insufficient breadth

□ Sufficient breadth but insufficient depth

□ Both are insufficient

1. Is the Seminar schedule reasonable (e.g., duration per session, frequency)?

□ Very reasonable

□ Reasonable

□ Moderately reasonable

□ Slightly unreasonable

□ Completely unreasonable

1. Sufficiency of instructor guidance (e.g., literature recommendations, methodological guidance)?

□ Very sufficient

□ Sufficient

□ Moderately sufficient

□ Insufficient

□ Very insufficient

1. Effectiveness of interaction formats (e.g., group discussions, online platforms)?

□ Very effective

□ Effective

□ Moderately effective

□ Slightly ineffective

□ Ineffective

V. Overall Satisfaction and Willingness for Improvement

1. Your overall satisfaction with the Seminar teaching model?

□ Very satisfied

□ Satisfied

□ Neutral

□ Dissatisfied

□ Very dissatisfied

1. Main advantages of the current model (multiple selections allowed):

□ Exposure to frontier knowledge

□ Facilitation of teacher-student communication

□ Enhancement of presentation skills

□ Stimulation of innovative thinking

□ Other ______

1. Main shortcomings of the current model (multiple selections allowed):

□ Limited topic selection

□ Monotonous interaction formats

□ Unreasonable time allocation

□ Inconsistent presentation quality

□ Other ______

1. Your specific suggestions for improving the Seminar teaching model: (Open-ended)

_______________________________________________________________________

1. Are you willing to continue participating in future Seminar activities?

□ Very willing

□ Willing

□ Neutral

□ Unwilling

□ Very unwilling

1. What additional content or formats would you like to see in future Seminars (e.g., inviting external experts, practical sessions)?

_______________________________________________________________________

End of Questionnaire. Thank you for your participation!
